# Joint Resource Allocation of Spectrum Sensing and Energy Harvesting in an Energy-Harvesting-Based Cognitive Sensor Network

**DOI:** 10.3390/s17030600

**Published:** 2017-03-16

**Authors:** Xin Liu, Weidang Lu, Liang Ye, Feng Li, Deyue Zou

**Affiliations:** 1 School of Information and Communication Engineering, Dalian University of Technology, Dalian 116024, China; zoudeyue@dlut.edu.cn; 2 College of Information Engineering, Zhejiang University of Technology, Hangzhou 310014, China; luweid@zjut.edu.cn (W.L.); fenglzj@zjut.edu.cn (F.L.); 3 Communication Research Center, Harbin Institute of Technology, Harbin 150080, China; yeliang@hit.edu.cn

**Keywords:** cognitive sensor, spectrum sensing, energy harvesting, resource allocation

## Abstract

The cognitive sensor (CS) can transmit data to the control center in the same spectrum that is licensed to the primary user (PU) when the absence of the PU is detected by spectrum sensing. However, the battery energy of the CS is limited due to its small size, deployment in atrocious environments and long-term working. In this paper, an energy-harvesting-based CS is described, which senses the PU together with collecting the radio frequency energy to supply data transmission. In order to improve the transmission performance of the CS, we have proposed the joint resource allocation of spectrum sensing and energy harvesting in the cases of a single energy-harvesting-based CS and an energy-harvesting-based cognitive sensor network (CSN), respectively. Based on the proposed frame structure, we have formulated the resource allocation as a class of joint optimization problems, which seek to maximize the transmission rate of the CS by jointly optimizing sensing time, harvesting time and the numbers of sensing nodes and harvesting nodes. Using the half searching method and the alternating direction optimization, we have achieved the sub-optimal solution by converting the joint optimization problem into several convex sub-optimization problems. The simulation results have indicated the predominance of the proposed energy-harvesting-based CS and CSN models.

## 1. Introduction

The cognitive sensor (CS), based on cognitive radio, has been proposed to transmit the sensing data using the idle spectrum licensed to the primary user (PU), when the PU is shortly absent. However, the CS must sense the absence of the PU by performing spectrum sensing before accessing the licensed spectrum [1,2]. During the transmission, the CS should sense the PU periodically. Once the presence of the PU is detected, the CS must vacate the licensed spectrum in order to avoid causing any harmful interference to the PU [3]. The spectrum sensing performance is reflected by false alarm probability and detection probability. The spectrum utilization improves with lower false alarm probability while the interference decreases with higher detection probability [4,5].

Energy detection has been proposed as an effective spectrum sensing method when the prior information of the detected signal is hard to obtain. However, energy detection performance may be degraded when the channel path between the PU and the CS is in severe fading or shadowing [6,7]. Hence, cooperative spectrum sensing has been proposed to improve the spectrum sensing performance for a cognitive sensor network (CSN), which allows multiple CSs to sense the PU collaboratively and uses a control center to combine the detection results of these CSs to make a final decision [8]. In [9], periodical cooperative spectrum sensing is proposed, where the sensing period is optimized to improve the spectrum utilization and reduce the interference. In [10], it has been proven that there is a sensing-throughput tradeoff in cognitive radio, i.e., there is an optimal sensing time that maximizes the transmission throughput.

Since the CS has limited battery energy due to its small size, deployment in atrocious environments and long-term working, its transmission performance is often restricted. Recently, energy harvesting has been proposed to collect and store the renewable radio frequency (RF) energies of the environmental signal sources. By deploying a rectifying circuit, the RF energy can be converted to the electrical power for supplying the transmission of a wireless communication system [11,12,13,14]. In a CSN, the CS may harvest the RF energy of the PU signal to improve transmission power when the PU is present in the channel [15,16,17]. In [18], an optimal energy-harvesting-based cooperative spectrum sensing model is proposed, which seeks to maximize the spectrum access probability of the CSN by jointly optimizing the sensing parameters such as sensing time and sensing node. However, the optimization of energy harvesting parameters such as harvesting time and harvesting node is not considered. Both sensing parameter and harvesting parameter have deep impacts on the transmission performance. In [19], energy-harvesting-aided spectrum sensing and data transmission is proposed, which minimizes the energy consumption by jointly allocating transmission time, transmission power and channels. However, the resources of spectrum sensing and data transmission are allocated independently in different sensors.

In this paper, a joint resource allocation of spectrum sensing and energy harvesting for an energy-harvesting-based CSN has been proposed, where spectrum sensing, energy harvesting and data transmission are synthetically considered in one CS. The contributions of the paper are listed as follows:

- We have investigated an energy-harvesting-based CSN model, which divides the CSs into two groups, one for sensing the PU cooperatively and the other one for harvesting the RF energy collaboratively. The harvested energy is used to increase the transmission energy of the CSN. Through considering spectrum sensing, energy harvesting and data transmission comprehensively, the transmission rate of the CS is improved while the sensing performance is guaranteed.
- We have investigated the joint resource optimization of sensing time, harvesting time and the numbers of sensing nodes and harvesting nodes. We have formulated the resource allocations of a single CS and CSN as two joint optimization problems, respectively. These optimization problems seek to maximize the transmission rate by obtaining a tradeoff between spectrum sensing and energy harvesting.

The rest of the paper is organized as follows. In Section 2, a single energy-harvesting-based CS together with its frame structure is described, and the joint time allocation of spectrum sensing and energy harvesting is performed. In Section 3, an energy-harvesting-based cooperative CSN including network structure and frame structure is presented, and the joint time-and-node allocation of spectrum sensing and energy harvesting is analyzed. The simulations and discussions are provided in Section 4. Finally, the conclusions are drawn in Section 5.

## 2. Resource Allocation in Single Cognitive Sensor

**Figure 1:**
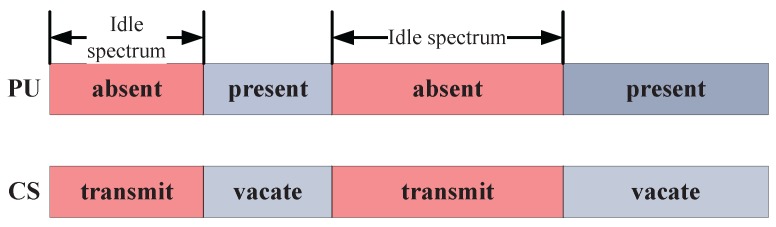
Idle spectrum utilization of the cognitive sensor.

The CSN is proposed based on the IEEE 802.22 defined as the cognitive radio wireless regional area network standard. In IEEE 802.22, the idle spectrum can be unused licensed channels over a given area, which can be reused by the CR network; however, if the spectrum availability changes, the 802.22 network must adapt quickly so as not to cause harmful interference to the licensed transmissions [20]. Thus, in the CSN, a CS may share the same spectrum with a PU in order to improve the spectrum utilization, but the CS cannot bring any interference to the PU when the PU is working. In this paper, the CS can only use the idle spectrum to transmit data when the PU is absent in the spectrum, however, the CS has to vacate the spectrum when the PU is present again, as shown in Figure 1. Thus, the CS can use the idle spectrum as a secondary user, who has to sense the absence of the PU before accessing the potentially idle spectrum. The limited energy of the CS may restrict the transmission performance especially when forwarding image or video data. In this section, the CS can harvest the RF energy of the outside signal sources before transmitting data, then the harvested energy is used to increase the transmission energy in order to guarantee the transmission quality.

### 2.1. Energy-Harvesting-Based Cognitive Sensor

**Figure 2:**
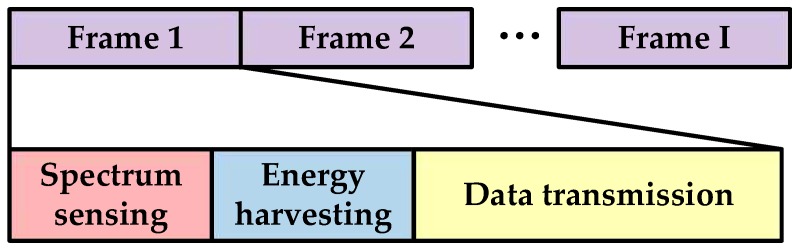
Frame structure of the single cognitive sensor.

We assume the CS performs spectrum sensing, energy harvesting and data transmission periodically. Thus, as shown in Figure 2, the frame structure of the CS is divided into three time slots: spectrum sensing slot, energy harvesting slot and data transmission slot. The CS senses the PU by energy detection in the spectrum sensing slot, while harvesting the RF energy in the energy harvesting slot. If the absence of the PU has been detected, the CS will forward data in the data transmission slot, otherwise the CS will stop communicating in the current frame and sense the PU again in the following frame. We suppose that the frame duration is *T*, the time length of the spectrum sensing slot is *τ* and the time length of the energy harvesting slot is *ε*. Then the time length of the data transmission slot is $T_{d}=T-\tau -\varepsilon$.

**Figure 3:**
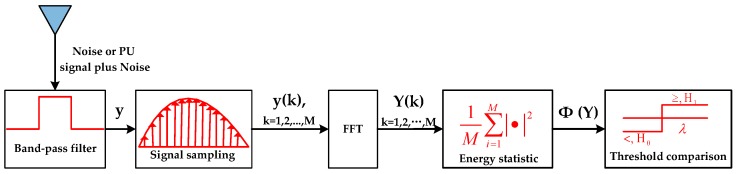
Energy detection structure.

Energy detection has been proposed as an effective spectrum sensing method, which can obtain an accurate detection result without needing any priori information of the detected signal. In the spectrum sensing slot, the CS uses energy detection to detect the presence of the PU. As shown in Figure 3, the energy detection process is described as follows. Firstly, We pass the received detected signal *y* through a band-pass filter to select an appropriate sensing frequency and sample’ /the received signal as y(k) using the sampling frequency $f_{s}$ within the sensing time *τ*; then, we get the frequency domain value of y(k) as Y(k) through the Fast Fourier Transformation (FFT) and obtain the energy statistic of the detected signal as Φ(Y) by averaging the squared amplitude of Y(k); finally, the energy statistic Φ(Y) is compared with a threshold *λ*. If Y≥λ, the presence of the PU, denoted by $H_{1}$, is decided, otherwise, the absence of the PU, denoted by $H_{0}$, is determined. The probabilities of $H_{0}$ and $H_{1}$ are assumed to be $P(H_{0})$ and $P(H_{1})$, respectively.

Thus, the detected signal is processed as a binary hypothesis problem as follows

(1) $$y\left(k\right)=\left\{\begin{array}{c} n\left(k\right),H_{0}\\ h\left(k\right)s\left(k\right)+n\left(k\right),H_{1} \end{array}\right.,k=1,2,...,M$$

where s(k) is the sampled PU signal with the power $p_{s}$, n(k) is the sampled Gaussian noise with the variance $\sigma_{n}^{2}$, h(k) is the channel gain between the PU and the CS, and $M=\tau f_{s}$ is the number of the sampling nodes. We suppose Y(k) for k=1,2,...,M are the FFT values of y(k) for k=1,2,...,M. The energy statistic of the detected signal is given by

(2) $$\Phi \left(Y\right)=\frac{1}{M}\sum_{k=1}^{M}{\left|Y\left(k\right)\right|}^{2}$$

Since Y(k) for k=1,2,...,M are independently and identically distributed, T(Y) obeys the Gaussian distribution with large *M* as

(3) $$\Phi \left(Y\right)∼\left\{\begin{array}{c} N\left(\sigma_{n}^{2},\frac{\sigma_{n}^{4}}{M}\right),H_{0}\\ N\left(\left(1+\gamma \right)\sigma_{n}^{2},\frac{{\left(1+\gamma \right)}^{2}\sigma_{n}^{4}}{M}\right),H_{1} \end{array}\right.$$

where N(a,b) denotes the Gaussian distribution with mean *a* and variance *b*, $\gamma =p_{s}h^{2}/\sigma_{n}^{2}$ is the PU signal to noise ratio (PSNR). By comparing Φ(Y) to a presettled decision threshold *λ*, the false alarm probability and detection probability of energy detection, related with sensing time and decision threshold, are given as [21]

(4) $$P_{f}\left(\tau ,\lambda \right)=P_{r}\left(\Phi \left(Y\right)>\lambda |H_{0}\right)=Q\left(\left(\frac{\lambda }{\sigma_{n}^{2}}-1\right)\sqrt{\tau f_{s}}\right)$$

(5) $$P_{d}\left(\tau ,\lambda \right)=P_{r}\left(\Phi \left(Y\right)>\lambda |H_{1}\right)=Q\left(\left(\frac{\lambda }{\sigma_{n}^{2}}-\gamma -1\right)\sqrt{\frac{\tau f_{s}}{{\left(\gamma +1\right)}^{2}}}\right)$$

where the function Q(x) is described as

(6) $$Q\left(x\right)=\frac{1}{\sqrt{2\pi }}\int_{x}^{+\infty }\exp\left(-\frac{z^{2}}{2}\right)\;dz$$

When the absence of the PU is detected accurately, the CS can transmit data effectively in the channel with the probability $\left(1-P_{f}\right)P\left(H_{0}\right)$. However, when the PU is present but the absence of the PU is detected falsely, the CS will also transmit data in the channel but cause harmful interference to the PU with the probability $\left(1-P_{d}\right)P\left(H_{1}\right)$. The interference power $p_{int}$ must be less than the maximal interference power $I_{max}$ suffered by the PU, as follows

(7) $$p_{int}=p_{t}h^{2}\left(1-P_{d}\right)P\left(H_{1}\right)\leq I_{max}$$

where $p_{t}$ is the transmission power produced by the original energy. Then we can get the lower limit of detection probability as

(8) $$P_{d}\geq P_{d}^{low}\;\;\mathrm{where}\;\;P_{d}^{low}=1-\frac{I_{max}}{P\left(H_{1}\right)p_{t}h^{2}}$$

From (4) and (5), *λ* can be obtained with the given $P_{d}^{low}$, then $P_{f}$ can be denoted by $P_{d}^{low}$ as follows

(9) $$P_{f}\left(\tau \right)\geq Q\left(Q^{-1}\left(P_{d}^{low}\right)\left(\gamma +1\right)+\gamma \sqrt{\tau f_{s}}\right)$$

**Figure 4:**
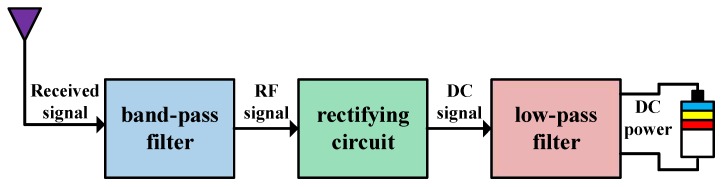
Energy harvesting process.

In the energy harvesting slot, the CS harvests the RF energy of the outside signal sources including the PU signal and the noise. As shown in Figure 4, the RF energy harvesting process is described as follows. We filter out the out-of-band electromagnetic radiation to the received signal through a band-pass filter, then convert the RF output of the filter to the DC signal by a rectifying circuit, and finally obtain the DC power by filtering out the harmonic and fundamental signals from the DC signal with a low-pass filter. However, some energy may be leaked to the surrounding environment due to the circuit electromagnetism compatibility, so we assume the energy harvesting efficiency is 0<μ<1. Noting that the PU appears with the probability $P(H_{1})$, the harvested energy of the CS within harvesting time *ε* is given by

(10) $$E_{h}=\mu \left(P\left(H_{1}\right)p_{s}h^{2}+\sigma_{n}^{2}\right)\varepsilon$$

$E_{h}$ is stored in a rechargeable battery and used to supply the transmission energy in the data transmission slot, thus the transmission power of the CS can be improved. Supposing τ≪T and ε≪T, the transmission power increment, related with *ε*, is given by

(11) $$\Delta p\left(\varepsilon \right)=\frac{\mu \left(P\left(H_{1}\right)p_{s}h^{2}+\sigma_{n}^{2}\right)}{T}\varepsilon$$

The transmission time of the CS within a frame is T−τ−ε, and the transmission probability of the CS is $\left(1-P_{f}\left(\tau \right)\right)P\left(H_{0}\right)$. The transmission rate of the CS is calculated from the Shannon–Hartley theorem as $W\log\left(1+\frac{\left(p_{t}+\Delta p\left(\varepsilon \right)\right)g^{2}}{\sigma_{n}^{2}}\right)$, where *W* is the bandwidth, and *g* is the channel gain between the CS and the control center. Thus, the average transmission rate of the CS is given by

(12) $$R\left(\tau ,\varepsilon \right)=\frac{T-\tau -\varepsilon }{T}\left(1-P_{f}\left(\tau \right)\right)P\left(H_{0}\right)\times W\log\left(1+\frac{\left(p_{t}+\Delta p\left(\varepsilon \right)\right)g^{2}}{\sigma_{n}^{2}}\right)$$

For simplification, we assume *W* = 1 kHz, which can be ignored in the following deductions.

### 2.2. Joint Time Resource Allocation

We seek to maximize the average transmission rate of the CS by jointly optimizing the spectrum sensing time and the energy harvesting time, subject to the constraint that the detection probability is above its lower limit, as follows

(13a) $$\begin{array}{cc} \max_{\tau ,\varepsilon }\;\; & R(\tau ,\varepsilon ) \end{array}$$

(13b) $$\begin{array}{cc} s.t.\;\; & P_{d}\geq p_{d}^{low} \end{array}$$

(13c) $$\begin{array}{cc} & \tau +\varepsilon \leq T \end{array}$$

(13d) $$\begin{array}{cc} & \tau \geq 0\;\;\mathrm{and}\;\;\varepsilon \geq 0 \end{array}$$

Since Q(x) is a monotonously decreasing function, from (4) and (5) both $P_{f}$ and $P_{d}$ decrease with the increasing of *λ*. Thus, to maximize *R*, $P_{f}$ should be decreased as low as possible, which indicates *λ* should be increased until $P_{d}$ achieves its lower limit, i.e., $P_{d}=p_{d}^{low}$. According to (9), the objective function of (13a) is rewritten as follows

(14) $$R\left(\tau ,\varepsilon \right)=\frac{T-\tau -\varepsilon }{T}\left(1-Q\left(Q^{-1}\left(P_{d}^{low}\right)\left(\gamma +1\right)+\gamma \sqrt{\tau f_{s}}\right)\right)P\left(H_{0}\right)\log\left(1+\frac{\left(p_{t}+\Delta p\left(\varepsilon \right)\right)g^{2}}{\sigma_{n}^{2}}\right)$$

Since (13) is a multi-parameter optimization problem, which is hard to be solved directly, we use alternating direction optimization (ADO) to obtain the sub-optimal solution [22]. Fixing *ε*, the optimization problem (13) converts to the sub-optimization problem about *τ* as follows

(15a) $$\begin{array}{cc} \max_{\tau }\;\; & R\left(\tau \right)=\frac{T_{0}-\tau }{T}B\left(1-P_{f}\left(\tau \right)\right)=\frac{T_{0}-\tau }{T}B\left(1-Q\left(A+\gamma \sqrt{\tau f_{s}}\right)\right) \end{array}$$

(15b) $$\begin{array}{cc} s.t.\;\; & 0\leq \tau \leq T_{0} \end{array}$$

where $T_{0}=T-\varepsilon$, $A=Q^{-1}\left(P_{d}^{low}\right)\left(\gamma +1\right)$ and $B=P\left(H_{0}\right)\log\left(1+\frac{\left(p_{t}+\Delta p\left(\varepsilon \right)\right)g^{2}}{\sigma_{n}^{2}}\right)$ can be seen as three constants with fixed *ε*. To solve (15), we give the following Theorem 1.

**Theorem** **1.** The problem (15) is a convex optimization problem about τ, i.e., there is a certain $\tau^{*}\in \left[0,\;\;T_{0}\right]$ that makes $R(\tau^{*})$ achieve the maximum.

**Proof** **of Theorem 1.** We calculate the first-order and secondary-order partial derivatives of $P_{f}\left(\tau \right)$ in *τ* as follows

(16) $$\nabla P_{f}\left(\tau \right)=-\frac{\gamma \sqrt{f_{s}}}{2\sqrt{2\pi \tau }}\exp\left(-\frac{{\left(A+\gamma \sqrt{\tau f_{s}}\right)}^{2}}{2}\right)$$

(17) $$\nabla^{2}P_{f}\left(\tau \right)=\frac{\gamma^{2}f_{s}}{4\sqrt{2\pi }\tau }\left(\frac{1}{\gamma \sqrt{\tau f_{s}}}+A+\gamma \sqrt{\tau f_{s}}\right)\exp\left(-\frac{{\left(A+\gamma \sqrt{\tau f_{s}}\right)}^{2}}{2}\right)$$

By letting $P_{f}\leq 0.5$, we have $A+\gamma \sqrt{\tau f_{s}}\geq 0$. Then from (16) and (17), we get $\nabla P_{f}<0$ and $\nabla^{2}P_{f}>0$, respectively. We also get $\nabla P_{f}\to -\infty$ with τ→0. We calculate the first-order and secondary-order partial derivatives of R(τ) in *τ* as

(18) $$\nabla R\left(\tau \right)=-\frac{B}{T}\left(1-P_{f}\right)-\frac{(T_{0}-\tau )B}{T}\nabla P_{f}$$

(19) $$\nabla^{2}R\left(\tau \right)=\frac{B}{T}\left(2\nabla P_{f}-(T_{0}-\tau )\nabla^{2}P_{f}\right)$$

Since 0≤Q(x)≤1, from (18) we have

(20) $$\begin{array}{cc} & \lim_{\tau \to 0}\nabla R\left(\tau \right)=-\frac{T_{0}B}{T}\lim_{\tau \to 0}\nabla P_{f}-\frac{B\left(1-Q(A)\right)}{T}\to +\infty \\ & \lim_{\tau \to T_{0}}\nabla R\left(\tau \right)=-\frac{B}{T}\left(1-Q(A+\gamma \sqrt{T_{0}f_{s}})\right)<0 \end{array}$$

which indicates there is a $\tau^{*}\in \left[0,T_{0}\right]$ that makes $\nabla R(\tau^{*})=0$. From (19), $\nabla^{2}R<0$ can be obtained with $\nabla P_{f}<0$ and $\nabla^{2}P_{f}>0$, which indicates that ∇R(τ) is a monotonously decreasing function. Hence, $\tau^{*}$ is the unique value that makes $R(\tau^{*})$ achieve the maximum. ☐

Obviously, ∇R(0)>0 and $\nabla R(T_{0})<0$. We can get the solution $\tau^{*}$ to (15) by the half searching algorithm, as shown in the Algorithm 1.
**Algorithm 1** Half searching algorithm for $\tau^{*}$
Initialize $\tau_{min}=0$, $\tau_{max}=T_{0}$ and the calculation precision *δ*;Set $\tau =\frac{\tau_{min}+\tau_{max}}{2}$;If ∇R(τ)≥0: let $\tau_{min}=\tau$;If ∇R(τ)<0: let $\tau_{max}=\tau$;Repeat step (2) to (4) until $|\tau_{min}-\tau_{max}|<\delta$;Output $\tau^{*}=\frac{\tau_{min}+\tau_{max}}{2}$.


Then fixing *τ*, the optimization problem (13) converts to the sub-optimization problem about *ε* as follows

(21a) $$\begin{array}{cc} \max_{\varepsilon }\;\; & R\left(\varepsilon \right)=\frac{T_{1}-\varepsilon }{T}C\log\left(1+\frac{\left(p_{t}+D\varepsilon \right)g^{2}}{\sigma_{n}^{2}}\right) \end{array}$$

(21b) $$\begin{array}{cc} s.t.\;\; & 0\leq \varepsilon \leq T_{1} \end{array}$$

where $T_{1}=T-\tau$, $C=\left(1-Q\left(Q^{-1}\left(P_{d}^{low}\right)\left(\gamma +1\right)+\gamma \sqrt{\tau f_{s}}\right)\right)P\left(H_{0}\right)$ and $D=\frac{\mu \left(P\left(H_{1}\right)p_{s}h^{2}+\sigma_{n}^{2}\right)}{T}$ are seen as three constants with fixed *τ*. To solve (21), we give the following Theorem 2.

**Theorem** **2.** The problem (21) is a convex optimization problem about ε, i.e., there is a certain $\varepsilon^{*}\in \left[0,\;\;T_{1}\right]$ that makes $R(\tau^{*})$ achieve the maximum.

**Proof** **of Theorem 2.** We calculate the first-order and secondary-order partial derivatives of R(ε) in *ε* as follows

(22) $$\nabla R\left(\varepsilon \right)=-\frac{C}{T}\log\left(1+\frac{\left(p_{t}+D\varepsilon \right)g^{2}}{\sigma_{n}^{2}}\right)+\frac{\left(T_{1}-\varepsilon \right)CDg^{2}}{T\left(\sigma_{n}^{2}+\left(p_{t}+D\varepsilon \right)g^{2}\right)}$$

(23) $$\nabla^{2}R\left(\varepsilon \right)=-\frac{2CDg^{2}}{T\left(\sigma_{n}^{2}+\left(p_{t}+D\varepsilon \right)g^{2}\right)}-\frac{\left(T_{1}-\varepsilon \right)CD^{2}g^{4}}{T{\left(\sigma_{n}^{2}+\left(p_{t}+D\varepsilon \right)g^{2}\right)}^{2}}$$

Since C<1, T≫1, T≫τ and T≫ε, from (22), we can get

(24) $$\begin{array}{cc} & \lim_{\varepsilon \to 0}\nabla R\left(\varepsilon \right)\approx \frac{CDg^{2}}{\sigma_{n}^{2}+p_{t}g^{2}}>0\\ & \lim_{\varepsilon \to T_{1}}\nabla R\left(\varepsilon \right)=-\frac{C}{T}\log\left(1+\frac{\left(p_{t}+DT_{1}\right)g^{2}}{\sigma_{n}^{2}}\right)<0 \end{array}$$

which indicates that there is a $\varepsilon^{*}\in \left[0,\;\;T_{1}\right]$ that makes $\nabla R(\varepsilon^{*})=0$. From (23), we may have $\nabla^{2}R\left(\varepsilon \right)<0$ with $\varepsilon \in [0,\;\;T_{1}]$. Hence, $R(\varepsilon^{*})$ can achieve the maximum. ☐

Similarly, we can get $\varepsilon^{*}$ by the half searching algorithm with the Algorithm 1. Then we use the ADO to solve the optimization problem (13) by repeating to optimize *τ* and *ε* alternatively by fixing one of these two parameters. The jointly optimal solution can be obtained until both *τ* and *ε* are convergent. The joint optimization of sensing time and harvesting time is shown in the Algorithm 2.
**Algorithm 2** Joint optimization algorithm based on ADO
Initialize the iteration index i=0, $\varepsilon^{\left(i\right)}$ with any fixed value within (0,T), $\tau^{\left(i\right)}=0$ and the calculation precision *δ*;With fixed $\varepsilon^{\left(i\right)}$, calculate $\tau^{*}$ by the half searching algorithm;Let $\tau^{\left(i+1\right)}=\tau^{*}$;With fixed $\tau^{\left(i+1\right)}$, calculate $\varepsilon^{*}$ by the half searching algorithm;Let $\varepsilon^{\left(i+1\right)}=\varepsilon^{*}$;Set i=i+1;Repeat step (2) to (6) until both $\tau^{\left(i\right)}$ and $\varepsilon^{\left(i\right)}$ are convergent;Output $\tau^{\left(i\right)}$ and $\varepsilon^{\left(i\right)}$.


## 3. Resource Allocation in Cooperative Cognitive Sensor Network

We consider a cooperative CSN consisting of *N* CSs and one control center. In the cooperative CSN, all the CSs will sense the PU and harvest the RF energy cooperatively; the control center manages the CSs and decides the available idle spectrum. Once the absence of the PU is detected, the CSN will access the spectrum to perform communications.

### 3.1. Energy-Harvesting-Based Cooperative Cognitive Sensor Network

**Figure 5:**
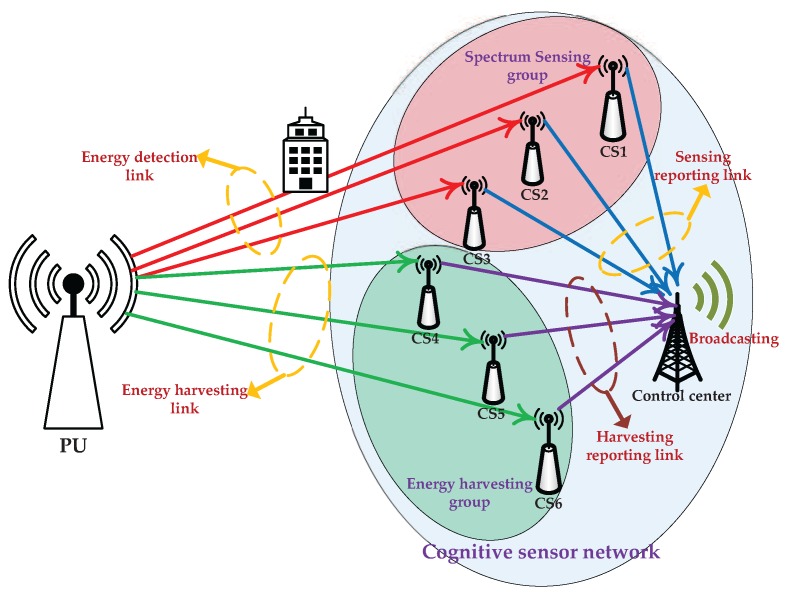
Cooperative cognitive sensor network structure.

**Figure 6:**
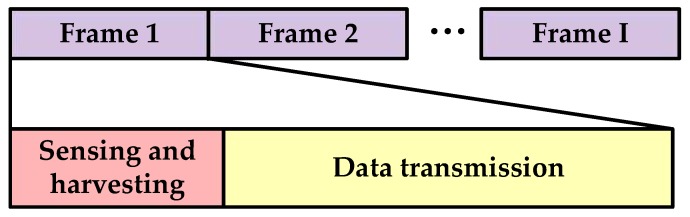
Frame structure of the cognitive sensor network.

We divide the *N* CSs into two groups. One group, namely the spectrum sensing group, senses the PU cooperatively, while the other group, namely the energy harvesting group, harvests the RF energy collaboratively, as shown in Figure 5. The frame structure of the CSN is divided into two time slots: sensing-and-harvesting slot and data transmission slot, as shown in Figure 6. In the sensing-and-harvesting slot, the spectrum sensing group and the energy harvesting group work simultaneously. If the absence of the PU is detected, the CSN will transmit data in the licensed spectrum with both the battery energy and the harvested energy.

**Figure 7:**
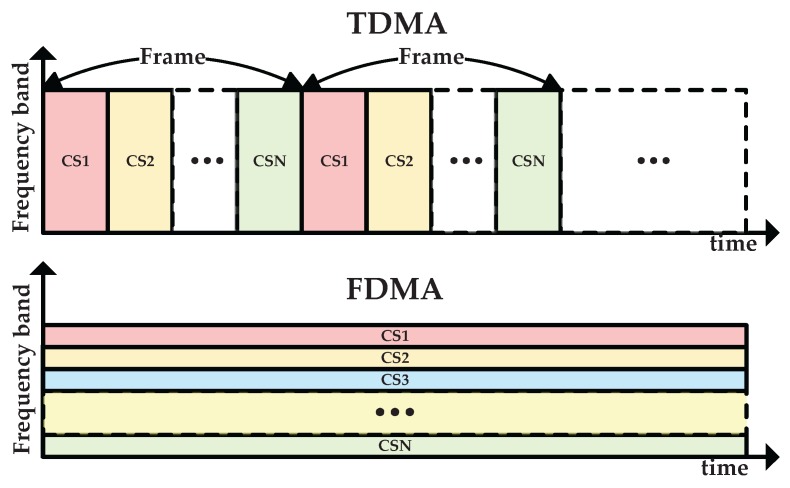
Frame structures of time division multiple access and frequency division multiple access.

The CSs in the sensing group perform cooperative spectrum sensing to improve the sensing performance. In the cooperative spectrum sensing, every CS senses the PU from the energy detection link and makes a local one-bit decision such as 0 (denoting $H_{0}$) or 1 (denoting $H_{1}$), then all the one-bit decisions are sent to the control center through the sensing reporting link, which are combined to get a final decision on the presence of the PU by “OR Rule” or “AND Rule” [23]. While the CSs in the harvesting group perform cooperative energy harvesting to collect the RF energy of the PU signal through the energy harvesting link. All the energy harvesting information is sent to the control center through the harvesting reporting link, which is used to allocate the harvested energy to each CS. The interactive sensing information and harvesting information is broadcasted to all the CSs by the control center. The CSs in the harvesting group will achieve the spectrum utilization state by receiving the broadcasted sensing information. Once the decision on the absence of the PU is achieved, each CS may have a chance to access the idle spectrum. To avoid causing harmful interference to each other, multiple CSs may transmit data by time division multiple access (TDMA) or frequency division multiple access (FDMA) in the data transmission slot. In the TDMA, the communication time is divided into several frames and each frame is further divided into several time slots, each CS transmits data in the allocated time slot periodically; while in the FDMA, the frequency band is divided into several subbands, each CS transmits data in the allocated subband, as shown in Figure 7.

The CS may have lower energy detection performance due to the channel fading or shadowing, however, by cooperative spectrum sensing, the detection performance in the fading channel can be improved by achieving sensing assistances from the other CSs. In OR-rule combination, the presence of the PU is finally decided if one CS has detected the presence of the PU. Thus, the OR-rule cooperative spectrum sensing can achieve higher detection probability compared with the single spectrum sensing. The false alarm probability and the detection probability of cooperative spectrum sensing are given as

(25) $$\Omega_{f}=1-{\left(1-P_{f,i}\right)}^{L}$$

(26) $$\Omega_{d}=1-{\left(1-P_{d,i}\right)}^{L}$$

where *L* is the number of the CSs in the spectrum sensing group, $P_{f,i}$ and $P_{d,i}$ are the probabilities of false alarm and detection of CS *i*, respectively. From (4) and (5), $\Omega_{f}$ can be denoted by fixing $\Omega_{d}=P_{d}^{low}$ as follows

(27) $$\Omega_{f}\left(\tau^{o}\right)=1-{\left(1-Q\left(Q^{-1}\left(1-{\left(1-P_{d}^{low}\right)}^{\frac{1}{L}}\right)\left(\gamma +1\right)+\gamma \sqrt{\tau^{o}f_{s}}\right)\right)}^{L}$$

where $\tau^{o}$ is the time length of the sensing-and-harvesting slot.

The number of the CSs in the energy harvesting group is N−L; the overall harvested energy of N−L CSs from the energy harvesting link is given as

(28) $$E_{h}^{o}\left(\tau^{o}\right)=\mu \left(N-L\right)\left(P\left(H_{1}\right)p_{s}h^{2}+\sigma_{n}^{2}\right)\tau^{o}$$

Hence, the total transmission power increment is given by $▵p\left(\tau^{o}\right)=\frac{E_{h}^{o}\left(\tau^{o}\right)}{T}=\left(N-L\right)D\tau^{o}$. Since the original transmission power of every CS is $p_{t}$, the total transmission power of the CSN after energy harvesting is $Np_{t}+\left(N-L\right)D\tau^{o}$. Since the time length of the data transmission slot is given by $T-\tau^{o}$, according to the theorem that $\frac{1}{N}\sum_{i=1}^{N}\log(1+x_{i})\leq \log\left(1+\frac{1}{N}\sum_{i=1}^{N}x_{i}\right)$, we can get the upper limit of the average overall transmission rate of the CSN as follows

(29) $$\begin{array}{cc} R^{o}\left(\tau^{o},L\right) & =\frac{T-\tau^{o}}{T}{\left(1-P_{f,i}\right)}^{L}P\left(H_{0}\right)\sum_{i=1}^{N}\log\left(1+\frac{p_{i}g^{2}}{\sigma_{n}^{2}}\right)\\ & \leq \frac{(T-\tau^{o})N}{T}{\left(1-P_{f,i}\right)}^{L}P\left(H_{0}\right)\log\left(1+\frac{\left(Np_{t}+\left(N-L\right)D\tau^{o}\right)g^{2}}{N\sigma_{n}^{2}}\right) \end{array}$$

### 3.2. Joint Time-And-Node Resource Allocation

Our goal is to maximize the average overall transmission rate of the CSN by jointly optimizing the spectrum sensing time and the number of CS nodes in the sensing group, subject to the constraint that the cooperative detection probability is above its lower limit, as follows

(30a) $$\begin{array}{cc} \max_{\tau^{o},L}\;\; & R^{o}\left(\tau^{o},L\right)=\frac{(T-\tau^{o})N}{T}{\left(1-P_{f,i}\left(\tau^{o}\right)\right)}^{L}P\left(H_{0}\right)\log\left(1+\frac{\left(Np_{t}+\left(N-L\right)D\tau^{o}\right)g^{2}}{N\sigma_{n}^{2}}\right) \end{array}$$

(30b) $$\begin{array}{cc} s.t.\;\; & \Omega_{d}\geq p_{d}^{low} \end{array}$$

(30c) $$\begin{array}{cc} & 0\leq \tau^{o}\leq T \end{array}$$

(30d) $$\begin{array}{cc} & 1\leq L\leq N,L\in Z \end{array}$$

Fixing *L*, the sub-optimization problem (30) converts to the optimization problem about $\tau^{o}$. According to the Theorem 1, we can easily know that the function $\phi \left(\tau^{o}\right)=\frac{(T-\tau^{o})N}{T}{\left(1-P_{f,i}\left(\tau^{o}\right)\right)}^{L}$ is convex in $\tau^{o}$. The function $\varphi \left(\tau^{o}\right)=\log\left(1+\frac{\left(Np_{t}+\left(N-L\right)D\tau^{o}\right)g^{2}}{N\sigma_{n}^{2}}\right)$ is also convex in $\tau^{o}$. Thus, we have $\nabla^{2}\phi \left(\tau^{o}\right)<0$ and $\nabla^{2}\varphi \left(\tau^{o}\right)<0$. Noting $\phi (\tau^{o})>0$, $\varphi (\tau^{o})>0$ and $\nabla \phi \left(\tau^{o}\right)\nabla \varphi \left(\tau^{o}\right)\leq 0$, we can get

(31) $$\nabla^{2}R^{o}\left(\tau^{o}\right)=\nabla^{2}\left(\phi \left(\tau^{o}\right)\varphi \left(\tau^{o}\right)\right)=\varphi \left(\tau^{o}\right)\nabla^{2}\phi \left(\tau^{o}\right)+\phi \left(\tau^{o}\right)\nabla^{2}\varphi \left(\tau^{o}\right)+2\nabla \phi \left(\tau^{o}\right)\nabla \varphi \left(\tau^{o}\right)<0$$

which indicates that $R^{o}\left(\tau^{o}\right)$ is also convex in $\tau^{o}$. Hence, the problem (30) is a convex optimization problem with fixed *L*. Moreover, $R^{o}\left(\tau^{o}\right)$ can achieve the maximum only when $\Omega_{d}=p_{d}^{low}$. By substituting (27), the objective function (30a) is calculated as

(32) $$\begin{array}{c} R^{o}\left(\tau^{o}\right)=\frac{(T-\tau^{o})N}{T}{\left(1-Q\left(Q^{-1}\left(1-{\left(1-P_{d}^{low}\right)}^{\frac{1}{L}}\right)\left(\gamma +1\right)+\gamma \sqrt{\tau^{o}f_{s}}\right)\right)}^{L}\times \\ P\left(H_{0}\right)\log\left(1+\frac{\left(Np_{t}+\left(N-L\right)D\tau^{o}\right)g^{2}}{N\sigma_{n}^{2}}\right) \end{array}$$

where we can also get the derivative $\nabla R^{o}\left(\tau^{o}\right)$. We can achieve the optimal ${\tau^{o}}^{*}$ by the half searching algorithm similarly with the Algorithm 1. Since *L* is an integer within 1 to *N*, it is not computationally complicated to get the optimal $L^{*}$ through the exhaustive searching as follows

(33) $$L^{*}=\underset{L=1,2,...,N}{\mathrm{argmax}}\;\;R^{o}\left({\tau^{o}}^{*},L\right)$$

## 4. Simulations and Discussions

The simulations are drawn using the Matlab Software, where the parameters are set in Table 1.

### 4.1. Single Cognitive Sensor Case

**Figure 8:**
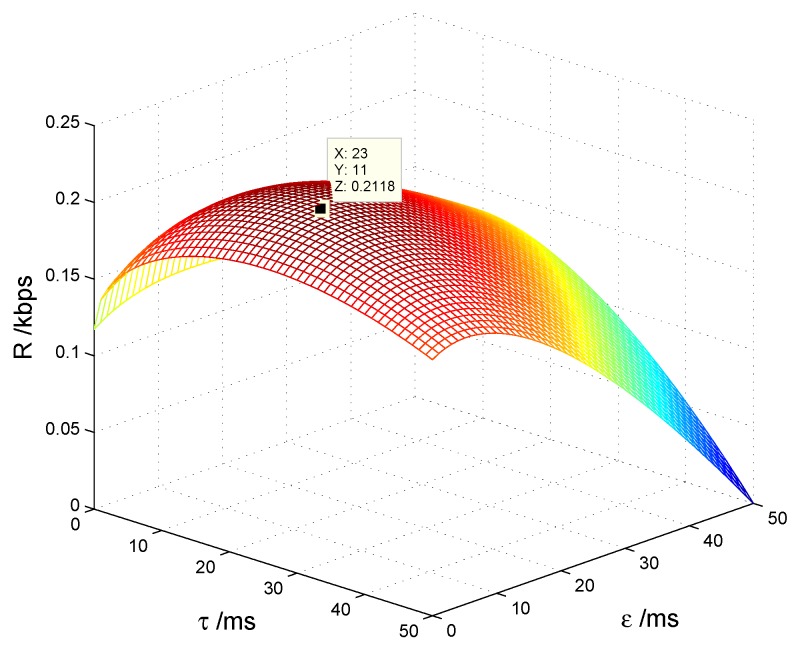
Transmission rate with spectrum sensing time and energy harvesting time.

In this section, we consider the single CS case. The channels obey the Rayleigh distribution with the gains changed according to the presettled PSNR. Figure 8 shows the average transmission rate of the CS *R* with different spectrum sensing time *τ* and energy harvesting time *ε*. We can see that there is an optimal set of *τ* and *ε* that maximizes *R*, i.e., the maximal R=0.2118 kbps when τ=23 ms and ε=11 ms.

**Figure 9:**
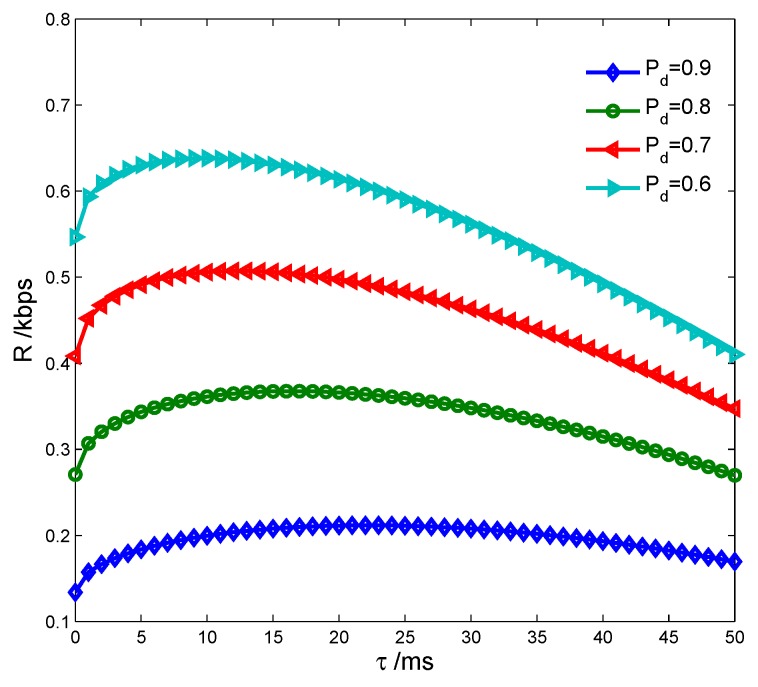
Transmission rate vs. detection probability with spectrum sensing time.

**Figure 10:**
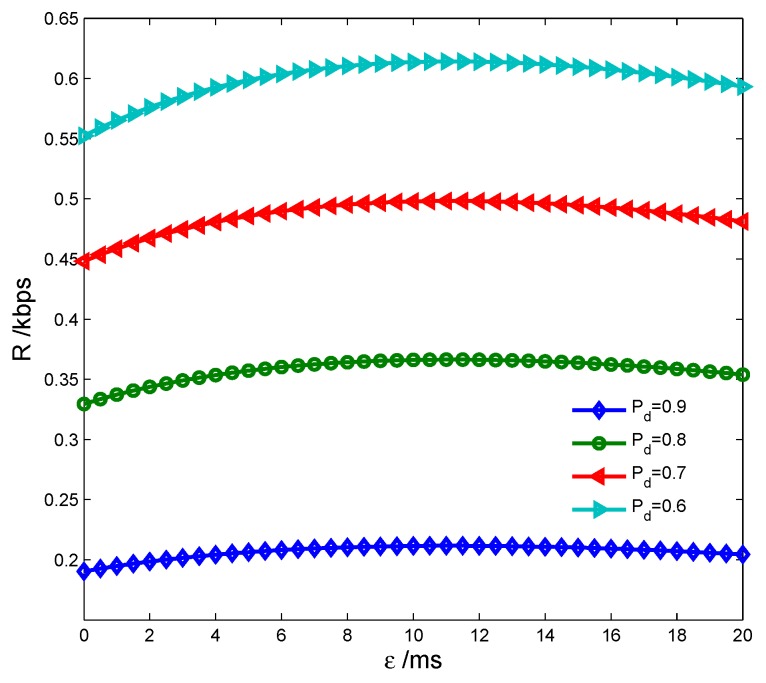
Transmission rate vs. detection probability with energy harvesting time.

Figure 9 shows the transmission rate vs. detection probability $P_{d}=\left\{0.9,0.8,0.7,0.6\right\}$ with different spectrum sensing time. We can see that *R* is lower both with less and larger *τ*, because the false alarm probability rises to decrease the spectrum access probability $\left(1-P_{f}\right)P\left(H_{0}\right)$ with less *τ*, while the sensing time increases to decrease the transmission time T−τ−ε with larger *τ*. Figure 10 indicates the transmission rate of the CS vs. detection probability with different energy harvesting time. It is seen that *R* is lower both with less and larger *ε*, because the transmission power increment ▵p(ε) is smaller with less *ε*, while the transmission time is shorter with larger *ε*. All these have proven the correctness of the Theorems 1 and 2. We also see that *R* improves as $P_{d}$ decreases, which indicates that *R* can achieve the maximum only when $P_{d}$ equals to the lower limit $P_{d}^{low}$.

**Figure 11:**
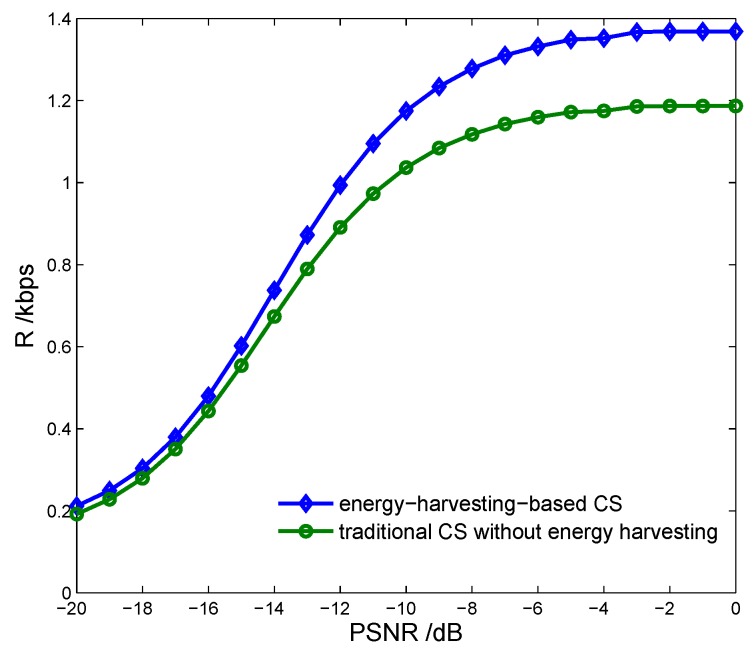
Transmission rate vs. cognitive sensor model with PU signal to noise ratio.

Figure 11 indicates the transmission rate of the CS vs. the proposed energy-harvesting-based CS model and the traditional CS model without energy harvesting [10]. We can see that the proposed energy-harvesting-based CS model results in a higher transmission rate than the traditional CS model, which indicates that energy harvesting can improve the transmission performance obviously.

### 4.2. Cognitive Sensor Network Case

**Figure 12:**
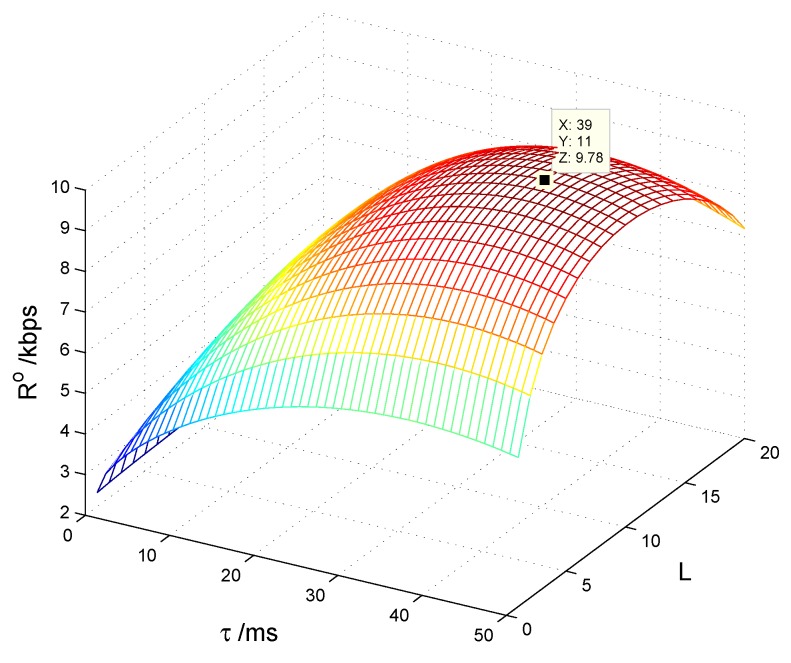
Transmission rate with spectrum sensing time and number of sensing cognitive sensors.

**Figure 13:**
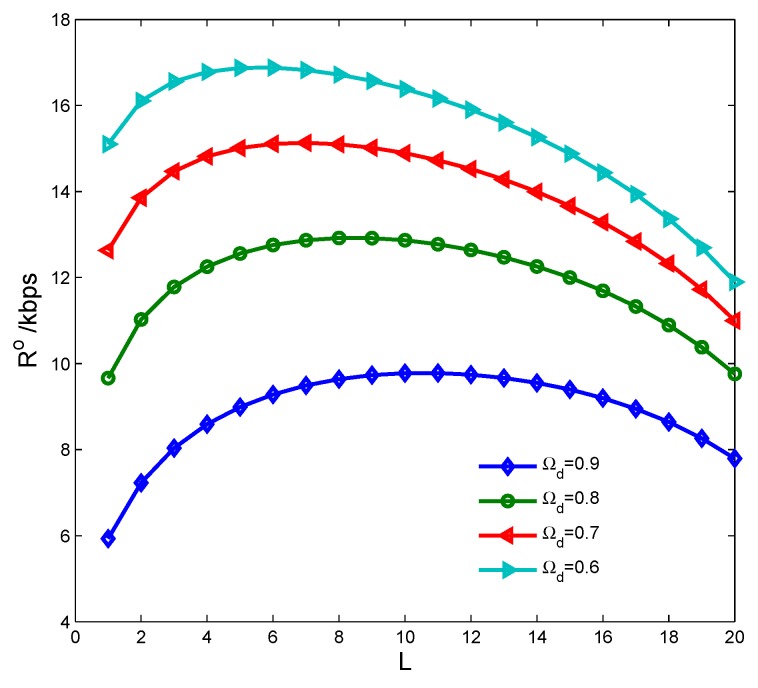
Transmission rate vs. cooperative detection probability with number of sensing cognitive sensors.

In this section, we consider the CSN case. The CSN is constituted of 20 CSs and one control center, where the CSs are divided into two groups, one group for cooperative spectrum sensing and the other one for cooperative energy harvesting; the control center manages all the CSs by broadcasting the decision information. Figure 12 shows the average overall transmission rate of the CSN $R^{o}$ with different spectrum sensing time $\tau^{o}$ and number of sensing CSs *L*. We can see that there is an optimal set of $\tau^{o}$ and *L* that makes $R^{o}$ achieve the maximum. Figure 13 shows the transmission rate of the CSN vs. cooperative detection probability $\Omega_{d}=\left\{0.9,0.8,0.7,0.6\right\}$ with different number of sensing CSs. It is seen that $R^{o}$ is lower both with smaller and larger *L*, because the false alarm probability increases to reduce the spectrum access with smaller *L*, while the harvested energy reduces to decrease the transmission power with larger *L*.

**Figure 14:**
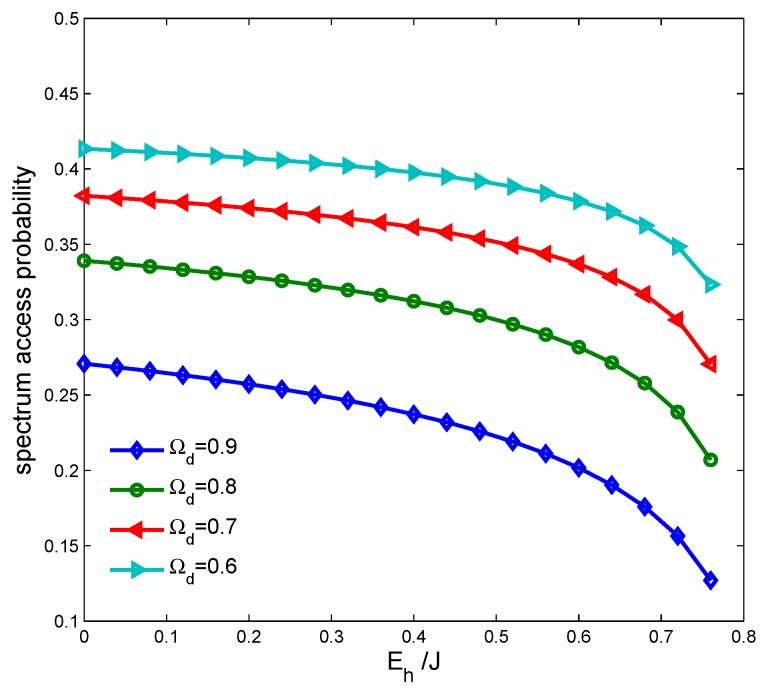
Spectrum access probability vs. cooperative detection probability with harvested energy.

**Figure 15:**
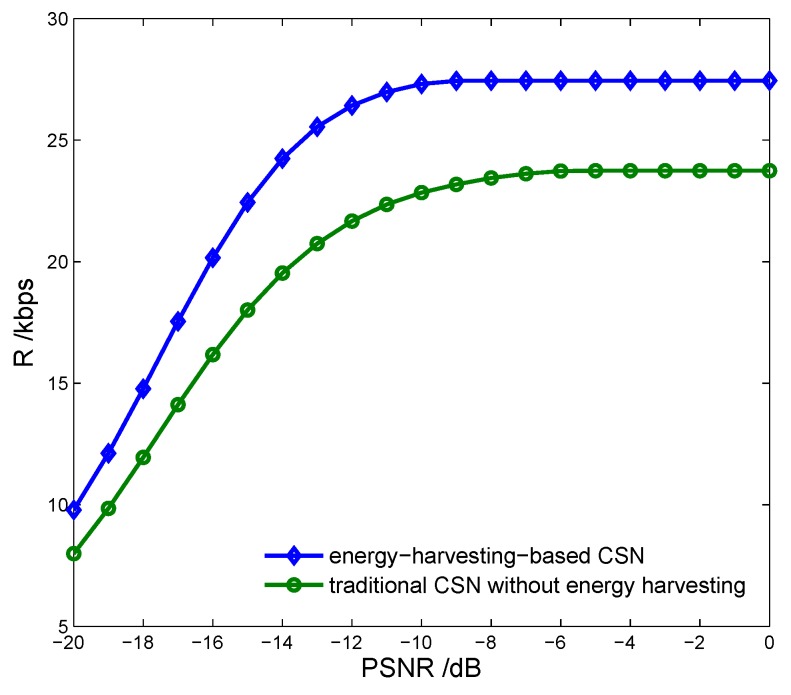
Transmission rate vs. cognitive sensor network model with PU signal to noise ratio.

Figure 14 indicates the spectrum access probability of the CSN, $\left(1-\Omega_{f}\right)P\left(H_{0}\right)$, vs. cooperative detection probability with different overall harvested energy $E_{h}^{o}$. We can see that the spectrum access probability decreases with the increasing of $E_{h}^{o}$. Thus, there is a tradeoff between improving the spectrum sensing and increasing the energy harvesting. In practice, we should consider improving the spectrum sensing preferentially in order to avoid causing any communication interference. Figure 15 shows the transmission rate of the CSN vs. the proposed energy-harvesting-based CSN model and the traditional CSN model without energy harvesting. We can see that the proposed model can achieve a higher transmission rate compared with the traditional model.

**Figure 16:**
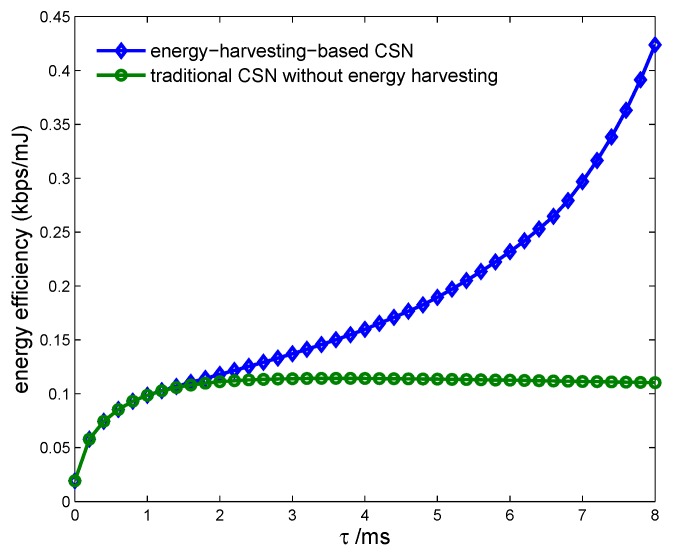
Energy efficiency vs. cognitive sensor network model with spectrum sensing time.

**Figure 17:**
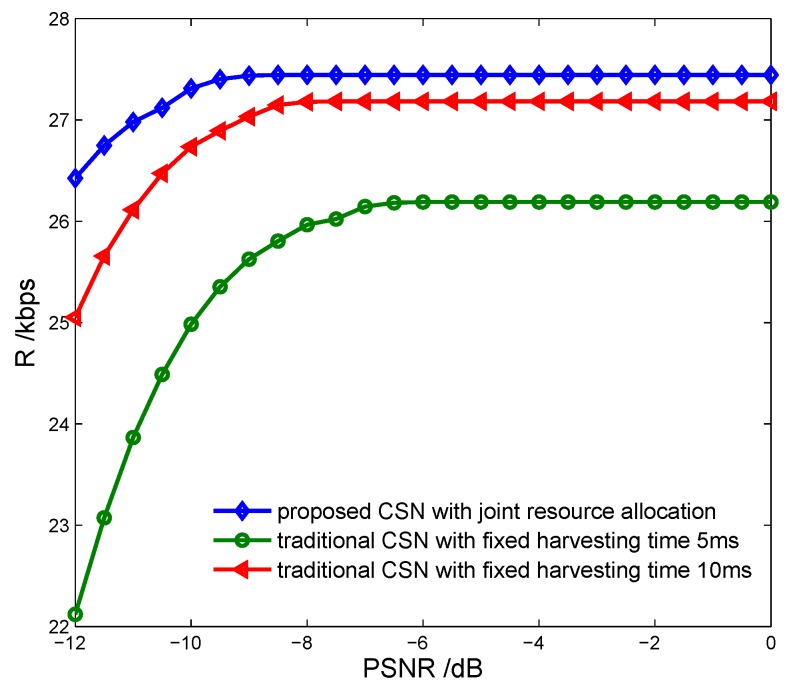
Transmission rate vs. cognitive sensor network model with fixed harvesting time.

Figure 16 shows the energy efficiency of the CSN vs. the proposed energy-harvesting-based CSN model and the traditional CSN model without energy harvesting, with different spectrum sensing time. We can see that the proposed model can achieve higher energy efficiency due to it harvesting more RF energy as the sensing time increases. Figure 17 indicates the transmission rate of the CSN vs. the proposed energy-harvesting-based CSN model with joint resource allocation and the traditional energy-harvesting-based CSN model with fixed harvesting time 5 ms and 10 ms. It is seen that the proposed model can achieve a higher transmission rate by optimizing the energy harvesting resources.

**Figure 18:**
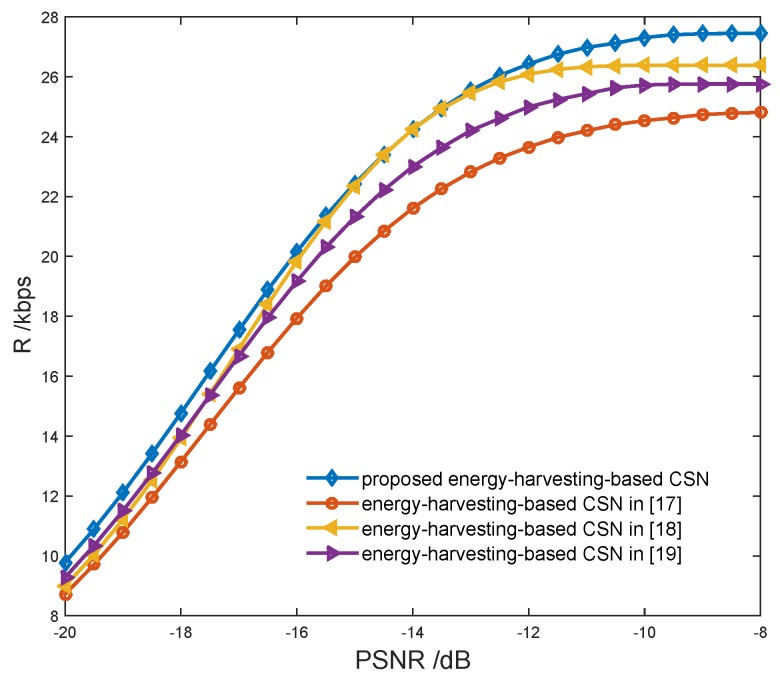
Transmission rate vs. energy-harvesting-based cognitive sensor network model.

In [17], energy harvesting is implemented only when the presence of the PU is detected, however, the noise energy can also be harvested when the PU is absent. In [18], the numbers of sensing nodes and harvesting nodes are presettled, hence, it is hard to achieve the optimal tradeoff between spectrum sensing and energy harvesting. In [19], the spectrum sensing and data transmission are implemented in different sensors; the CSs in the sensing group cannot transmit data even when the PU is absent. In the proposed model, both the energy of the PU signal and the noise can be harvested; the optimal joint resource allocation of spectrum sensing and energy harvesting is achieved to improve the transmission performance while guaranteeing the sensing performance, and all the CSs in the CSN can transmit data with TDMA or FDMA when the PU is absent. As shown in Figure 18, the proposed energy-harvesting-based CSN model can achieve higher throughput compared with the CSN models in [17,18,19].

## 5. Conclusions

In this paper, we have proposed the joint resource allocation of spectrum sensing and energy harvesting in a single CS and a CSN, respectively. The frame structures of energy-harvesting-based CS and CSN models are presented to allocate the communication resources. We have formulated the joint resource allocation as joint optimization problems, which seek to maximize the transmission rates of the CS and CSN by jointly optimizing the sensing time, the harvesting time and the numbers of sensing nodes and harvesting nodes. Based on the ADO, we have converted the joint optimization problem into several sub-optimization problems and proven the convex characteristics of these sub-optimization problems. From the simulation results, we have drawn the following conclusions: (1) there is an optimal set of spectrum sensing and energy harvesting parameters that maximizes the transmission rates of CS and CSN; (2) there is a tradeoff between improving spectrum sensing and increasing energy harvesting; (3) the proposed energy-harvesting-based CS and CSN models outperform the traditional models.

## Figures and Tables

**Table 1:** Simulation parameters setting.

Parameters	Values
Frame duration *T*	100 ms
Sampling frequency $f_{s}$	100 kHz
Absence probability of PU $P(H_{0})$	0.5
Presence probability of PU $P(H_{1})$	0.5
Transmission power of CS $p_{t}$	1 mW
Noise power $\sigma_{n}^{2}$	$10^{-3}$ mW
Transmission power of PU $p_{s}$	3 W
Energy harvesting efficiency *μ*	0.6
Number of CSs *N*	20

## Abbreviations

The following abbreviations are used in this manuscript:

CS	Cognitive Sensor
CSN	Cognitive Sensor Network
PU	Primary User
PSNR	PU signal to noise ratio
ADO	Alternating direction optimization
